# Cumulative ecological risk, dysfunctional beliefs and attitudes about sleep, and sleep quality among Chinese adolescents: the indirect association of psychological distress

**DOI:** 10.3389/fpsyg.2026.1821147

**Published:** 2026-09-14

**Authors:** Hongru Jia, Yongkang Cheng

**Affiliations:** College of Educational Science, Henan Finance University, Zhengzhou, Henan, China

**Keywords:** adolescents, cumulative ecological risk, dysfunctional beliefs and attitudes about sleep, psychological distress, sleep quality

## Abstract

**Background:**

Adolescent sleep problems have gained increasing attention; however, systematic research integrating both external environmental and internal cognitive factors is still lacking. This study focuses on Chinese adolescents and investigates the associations among cumulative ecological risk, dysfunctional beliefs and attitudes about sleep, psychological distress, and sleep quality. The goal is to examine the potential indirect associations involving psychological distress within the context of cognitive and environmental factors.

**Methods:**

This study employed a random sampling method to survey 793 Chinese middle and high school students. Standardized instruments were employed to assess students’ cumulative ecological risk, dysfunctional beliefs and attitudes about sleep, psychological distress, and sleep quality.

**Results:**

An indirect association model was constructed and tested. Both cumulative ecological risk and dysfunctional beliefs and attitudes about sleep were negatively associated with sleep quality, and psychological distress was statistically involved in the indirect associations between each risk factor and sleep quality. The estimated indirect association involving psychological distress represented 52.27% of the overall association between cumulative ecological risk and sleep quality and 36.79% of the overall association between dysfunctional beliefs and attitudes about sleep quality.

**Discussion:**

This study incorporates both cumulative ecological risk and dysfunctional beliefs and attitudes about sleep into a unified model, indicating a significant indirect association involving psychological distress. It highlights meaningful connections among environmental risks, cognitive biases, psychological distress, and sleep quality. These outcomes broaden our comprehension of the elements relating to teenage sleep issues, while simultaneously offering both theoretical backing and practical guidance for psychological services in schools, family-based intervention efforts, and the formulation of relevant policies.

## 1 Introduction

In the context of ongoing social changes and increasing educational pressures, adolescent sleep health issues have garnered widespread attention. Recent research suggests that around 26% of Chinese adolescents experience sleep disorders, and this proportion appears to be even greater among high school students. This trend highlights the heightened occurrence of sleep-related difficulties during academically demanding phases (Liang et al., 2021). Sleep, as a fundamental process related to energy restoration and the regulation of physiological rhythms, is closely associated with adolescents’ attention, emotional stability, cognitive flexibility, learning efficiency, and psychosocial functioning (Wang W. J. et al., 2024; Xu et al., 2025; Yuan et al., 2023). Therefore, exploring the current state of adolescent sleep quality (SQ) and its associated factors is of theoretical and practical relevance for advancing the understanding of adolescents’ psychological adaptation and mental health conditions.

In recent years, researchers have increasingly emphasized the role of environmental stressors and cognitive traits in understanding factors associated with sleep difficulties. Among these, cumulative ecological risk (CER) is seen as a comprehensive indicator of the multiple environmental burdens individuals face, reflecting cumulative exposure to risk factors from family, school, peers, and community (Evans et al., 2013). Adolescents living in high ecological risk environments over long durations tend to report more frequent negative emotional experiences and greater challenges in adaptation (Sun et al., 2021; Wang J. J. et al., 2024). Beliefs concerning sleep may be related to how individuals interpret their nightly rest and are closely associated with subjective perceptions of sleep. Dysfunctional beliefs and attitudes about sleep (DBAS) reflect exaggerated and catastrophic cognitive tendencies regarding sleep processes, sleep efficiency, and its consequences. These beliefs are common in adolescent populations and may be linked to sleep anxiety and reduced emotional resilience (Morin et al., 2007; Peng et al., 2023; Wang et al., 2025). Furthermore, psychological distress (PD), a non-specific negative emotional state often manifested as anxiety, depression, and irritability, is considered an important psychological response variable associated with environmental and cognitive factors. It is also widely regarded as an important psychological factor associated with SQ (AlRasheed et al., 2022; Duong, 2021; Kessler et al., 2002; Li et al., 2023).

Although previous studies have explored the relationships between CER, DBAS, or PD and SQ, there is still limited research that incorporates both ecological stress and cognitive biases in a unified model to examine the potential indirect associations among these variables involving emotional factors in relation to SQ. Specifically, there is a lack of systematic modeling and empirical validation in adolescent populations. Drawing upon the Ecological Systems Theory (EST) and the Stress-Vulnerability Model (SVM), whether psychological distress is involved in these associations that incorporates CER, DBAS, PD, and SQ to explore the relationships among these constructs within the identified research context. The goal is to examine whether multi-source ecological risks and cognitive biases are statistically associated with adolescents’ subjective sleep experiences, and whether psychological distress is involved in these associations. By shedding light on the interrelated factors linked to adolescent sleep challenges, this work outlines conceptual foundations and actionable suggestions for implementing multifaceted strategies. These approaches emphasize the joint application of ecological frameworks and cognitive models in advancing mental health initiatives.

## 2 Theory and hypotheses

### 2.1 Theoretical framework

The present research is grounded in EST and SVM frameworks and investigates the relational dynamics among CER, DBAS, and SQ in adolescents, examining the indirect association involving PD within the proposed model.

Bronfenbrenner (1979) framework, known as Ecological Systems Theory (EST), conceptualizes development as a dynamic process occurring within several interrelated environmental layers. The ecological framework includes four nested systems: the microsystem, referring to close environments including home and school; the mesosystem, which captures the relationships among these immediate contexts; the ecosystem, comprising more distal influences like neighborhood institutions; and the macrosystem, which reflects the values, norms, and cultural ideologies that shape an individual’s broader social world. From this theoretical perspective, environmental risks are viewed as interconnected rather than independent, and their aggregation over time is often linked to variations in individuals’ emotional functioning and behavioral patterns. Researchers have increasingly referred to CER as a way to characterize the combined presence of multiple adversities encountered by adolescents in various settings. These include difficulties in peer relationships, breakdowns in family functioning, heightened school-related demands, and limited protection or support within the local environment. The cumulative exposure to these risk factors is seen as a significant indicator of the challenges adolescents face in their developmental trajectory (Evans et al., 2013) and is thought to be associated with disturbances in emotional regulation, persistent PD, and poorer sleep health (Liu et al., 2024; Mang et al., 2023; Xie et al., 2024).

Nevertheless, although EST offers a conceptual model for comprehending the ways in which environmental stress relates to individual development, it has rarely addressed the role of an individual’s inherent psychological traits. To better understand how individuals respond differently to ecological risk, this study introduces SVM as a complementary perspective. Originally introduced by Zubin and Spring (1977), this model posits that psychological difficulties may emerge when environmental stressors intersect with an individual’s underlying susceptibilities. Environmental stress can arise from family, school, or social systems, while individual vulnerability manifests in cognitive biases, personality traits, or physiological sensitivity. In this study, DBAS are considered a cognitive susceptibility factor. Research has shown that adolescents who consistently hold exaggerated expectations, erroneous attributions, or catastrophic explanations about sleep typically report higher levels of PD (such as anxiety and depression) and also experience poorer SQ (Faaland et al., 2023; Morin et al., 2007; Wang et al., 2025).

In summary, EST and SVM together form the theoretical foundation of this study. The former emphasizes the associations between multiple environmental risks and adolescent mental health, while the latter provides a framework for examining whether PD is involved in the association between internal cognitive susceptibility and environmental stress. By integrating these two theoretical perspectives, this study aims to test a mediation model examining the indirect associations among CER, DBAS, PD, and SQ. The findings may provide theoretical references for sleep health–related practice and policy considerations.

### 2.2 Hypotheses

#### 2.2.1 Cumulative ecological risk and sleep quality

The concept of CER captures the accumulation of environmental adversities experienced by individuals, which may involve interpersonal tensions, disrupted family dynamics, school-related demands, and inadequate protective conditions in the broader neighborhood. Across developmental stages, environmental risks frequently emerge in clusters and intensify over time. Their cumulative presence has been linked to challenges in both bodily health and emotional functioning during adolescence (Bronfenbrenner, 1977). Compared to a single risk factor, CER provides a more comprehensive reflection of the overall burden and complexity of the environment that adolescents face.

Prior research has indicated that CER is significantly associated with variations in SQ among adolescents (Mang et al., 2023; Williamson et al., 2021; Xie et al., 2024). Xie et al. (2024) investigated the relationships between sleep quality and stressors originating from family life, academic demands, and peer interactions in a sample of first-year college students. Their results suggest that CER may be associated with individual differences in sleep conditions. Their findings indicated that college freshmen are generally exposed to a range of risks, and higher levels of CER are associated with poorer SQ. Williamson et al. (2021) investigated how various cumulative socio-demographic factors, such as family economic conditions and parents’ educational background, were related to sleep difficulties in young children. The study reported that increased accumulation of risk factors corresponded with lower SQ and more problematic sleep behaviors. This study provides an important context for understanding the complex relationship between ecological risk environments and sleep health across different developmental stages. Additionally, Mang et al. (2023) noted that in today’s rapidly changing social context, the sources of ecological risk faced by adolescents are increasingly diversified, such as experiences of social exclusion and exposure to cyberbullying. These unresolved risks are often associated with elevated psychological burdens and poorer SQ. In summary, there may be a significant relationship between CER and SQ, a relationship that spans different developmental stages and is prevalent in various social contexts. The co-existence of multiple risk factors reflects the complexity of an individual’s ecological environment and underscores the sensitivity of SQ to environmental stress.

#### 2.2.2 Dysfunctional beliefs and attitudes about sleep and sleep quality

Dysfunctional beliefs and attitudes about sleep refer to a set of inaccurate, irrational, and disruptive thoughts, beliefs, and attitudes about sleep commonly held by individuals with insomnia (Morin et al., 2007). Among those affected by insomnia, distorted cognitive patterns appear to be common and may relate to ongoing challenges in initiating and maintaining healthy SQ.

Existing literature has demonstrated a significant relationship between DBAS and SQ (Ballot et al., 2021; Karsan et al., 2024; Kuhn et al., 2024; Peng et al., 2023; Sullivan et al., 2022; Wang et al., 2025). Peng et al. (2023) focused on COVID-19 patients receiving treatment in makeshift hospitals during the pandemic and found a high prevalence of DBAS in this group. Individuals holding such beliefs generally showed poorer SQ. Within the specific circumstances of a widespread health emergency, the research pointed to a notable link between individuals’ DBAS and their perceived SQ. Wang et al. (2025) conducted a study among Chinese university students, exploring factors associated with SQ. Their results indicated a negative correlation between DBAS and SQ may have potential indirect links via negative feelings like stress and anxiety. Sullivan et al. (2022) extended this research to a broader population, examining how DBAS manifest across different socio-cultural contexts and their association with SQ, thus offering a cross-cultural perspective on the phenomenon. In summary, DBAS are consistently associated with SQ across different populations and contexts. Among youth navigating major physical and emotional transitions, this link stands out as especially relevant. Adolescents are highly susceptible to cognitive biases and emotional fluctuations, and their sleep beliefs are likely to be closely linked to their subjective sleep experiences and behaviors. Understanding how young individuals perceive sleep and how these perceptions correlate with SQ offers an opportunity to reflect on underlying psychological dynamics, potentially informing future efforts in psychoeducational planning and cognitive reframing.

#### 2.2.3 The indirect association of psychological distress

Psychological distress encompasses a wide range of unpleasant emotional experiences such as anxiety, low mood, frustration, helplessness, emotional tension, and irritability. These responses commonly accompany individuals’ attempts to manage psychological strain associated with internal or external challenges. This state can be short-term or persist over time and may be associated with differences in an individual’s daily functioning and quality of life (Kessler et al., 2002).

At the same time, increasing empirical evidence has validated the strong connection between PD and SQ (AlRasheed et al., 2022; Duong, 2021; Lee et al., 2025; Li et al., 2023; Xu et al., 2024). AlRasheed et al. (2022) conducted a study during the COVID-19 pandemic among the general population in Saudi Arabia and observed that individuals reporting higher PD also tended to experience lower SQ. The study draws attention to the intertwined presence of negative emotional states and sleep related difficulties that emerged during a large-scale public health emergency. Li et al. (2023) further noted that in adolescent populations, PD associated with peer “head-down behaviors” was significantly related to subjective SQ. This relationship was often co-occurring with problematic smartphone use, forming a multifaceted psychological-behavioral pattern associated with SQ. Duong (2021) also found in a study of Vietnamese adults that PD was significantly associated with subjective experiences of sleep difficulties and reduced attention. This finding suggests that the relationship between PD and SQ is worthy of continued attention, particularly in the face of social environmental stress. In summary, existing research across different socio-cultural contexts has identified a potential relationship between PD and SQ. Adolescents, as a group in the process of physical and psychological development, are more sensitive to environmental pressures and emotional fluctuations, and their SQ may be closely associated with psychological states. Therefore, examining the relationship between PD and SQ in adolescence has theoretical and practical value for identifying potential mental health risk patterns and informing developmental support strategies related to adaptation and well-being.

Existing research has increasingly highlighted the relationship between CER and PD (Liu et al., 2024; Sun et al., 2021; Wang J. J. et al., 2024). Liu et al. (2024) in their investigation involving Chinese adolescents, reported a significant association between CER and depressive symptoms. Those experiencing high CER appeared more vulnerable to emotional disturbances and psychological strain under conditions of elevated environmental stress. This finding further supports the potential close relationship between CER and PD. Sun et al. (2021) observed that when adolescents encountered multiple adverse life conditions, such as strained family dynamics, intense academic stress, or interpersonal hostility, they frequently reported symptoms aligned with depression, anxiety, and stress, reflecting characteristics of PD. Their study emphasized that in environments with CER, individuals are more likely to experience emotional fluctuations and adaptation difficulties, further supporting the association between CER and PD. Wang J. J. et al. (2024) found evidence that adolescents exposed to greater CER exhibited a higher prevalence of psychological distress–related behaviors, including suicidal ideation, planning, and acts of self-injury without suicidal intent. The connection between CER and individuals’ negative emotional experiences and mental conditions has been observed in prior findings, indicating a potential association worth further exploration. The study highlighted that higher exposure to multidimensional social-ecological risks was associated with higher reported levels of PD among adolescents. Therefore, PD may represent a potential indirect pathway between CER and SQ, providing a new theoretical perspective for understanding how multiple environmental pressures are related to individuals’ sleep experiences. Examining this indirect association contributes to a more systematic analytical model and may inform future research on multidimensional support strategies related to adolescents’ emotional regulation and sleep health.

Furthermore, there is a notable relationship between DBAS and PD (Faaland et al., 2023; Peng et al., 2023; Wang et al., 2025). From a cognitive-behavioral perspective, Faaland et al. (2023) observed that engagement in digital insomnia-focused CBT was linked to decreased psychological distress and a transition toward healthier sleep-related cognitions. Peng et al. (2023) further reported that more than half of the patients in a COVID-19 makeshift hospital exhibited varying degrees of DBAS, which were significantly associated with higher levels of psychological distress, including anxiety, depression, and insomnia. The study emphasized that correcting irrational beliefs about sleep may be one of the key interventions for alleviating PD during the pandemic. Wang et al. (2025) noted a connection between DBAS and heightened emotional responses like anxiety and stress. These emotional patterns are frequently present in individuals experiencing PD. This suggests a close relationship between DBAS and PD. In summary, the relationship between DBAS and PD has been consistently observed in multiple studies. PD may be statistically involved in the indirect association between DBAS and SQ, providing insights into the relationships among cognition, emotion, and sleep states. Exploring this connection will help enrich the theoretical perspectives on adolescent sleep research and provide an experiential foundation for future intervention designs.

In summary, although existing studies have separately explored the relationships between CER, DBAS, and adolescent SQ, and have preliminarily identified associations involving PD in this process, there is still a lack of systematic theoretical integration and comprehensive analysis of the associations among these three factors in relation to SQ. To address this gap, this study constructs a mediation model (see Figure 1) based on EST and the SVM, systematically testing the indirect associations among CER, DBAS, PD, and SQ. This model contributes to understanding the factors associated with adolescent sleep problems and may offer theoretical and empirical references for future multilevel mental health research and practice. The research hypotheses are as follows:

**Figure 1:**
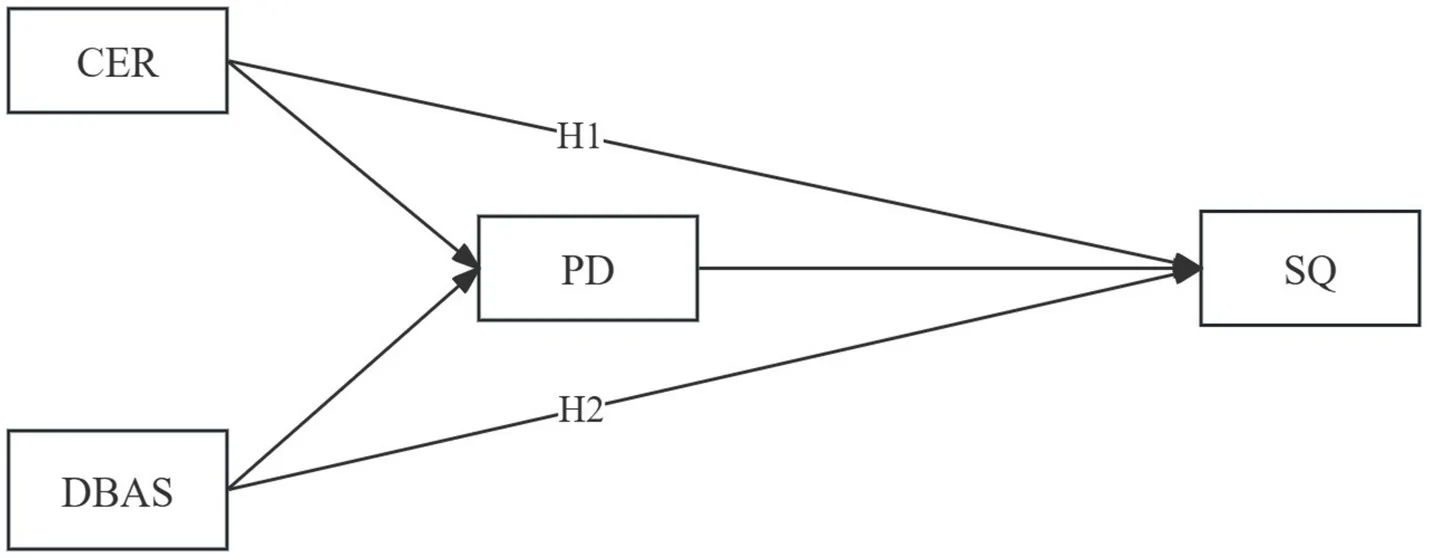
Research hypothesis model.

*H1*: CER is negatively associated with SQ.

*H2*: DBAS are negatively associated with SQ.

*H3*: PD is involved in the indirect association between CER and SQ.

*H4*: PD is involved in the indirect association between DBAS and SQ.

## 3 Research methods

### 3.1 Sample and data collection procedures

The study was conducted using an online questionnaire distributed through the Wenjuanxing platform^1^ between May and June 2025. Data were collected from three middle and high schools located in Henan Province in China. Before data collection, the research team contacted the relevant school administrators, explained the purpose and procedures of the study, and obtained permission to conduct the survey. To protect institutional anonymity, the names of the participating schools are not reported.

The sampling frame consisted of students aged 12–18 years who were formally enrolled in the participating schools during the data collection period and whose names appeared on the official class rosters. A school-based sampling strategy with simple random selection of students within each participating school was used. In each school, the official class rosters of eligible students were used to construct a school-specific sampling frame. Each eligible student was assigned a unique numerical code, and computer-generated random numbers were then used to select students without replacement. The random-selection procedure was implemented independently in each participating school.

After the random selection had been completed, class advisors contacted the selected students during scheduled class meetings and invited them to participate. The students were informed of the study purpose, survey procedures, voluntary nature of participation, and principles of confidentiality. They were also informed that they could decline or withdraw from participation without any negative consequences. For participants under 18 years of age, informed consent was obtained from their parents or legal guardians, and the students provided assent before completing the questionnaire. Participants aged 18 years provided their own informed consent. The questionnaire link or QR code was provided only to the randomly selected students who agreed to participate and was not publicly distributed to other students. All submitted responses were treated as strictly confidential and used solely for academic research.

Kline (2018) recommends including at least 10 participants for each item when determining sample size. With a total of 62 items in the current questionnaire, and considering an expected attrition rate of 20%, the minimum required sample was projected to be 744. To meet this threshold, 825 surveys were distributed, all of which were returned. After evaluating the responses, 32 were excluded due to issues such as missing data or consistently extreme answers across all items. The remaining 793 valid questionnaires were used in subsequent statistical analyses.

As summarized in Table 1, the sample included 393 males (49.6%) and 400 females (50.4%). A total of 589 participants (74.3%) were between 16 and 18 years old, and 583 (73.5%) were enrolled in high school. Urban residents accounted for 529 participants (66.7%), while 506 individuals (63.8%) were day students. Statistical analysis revealed no significant differences in SQ across gender, age, grade level, residential background, or boarding status (all *p*-values > 0.05).

**Table 1:** Demographic characteristics of participants.

Variable	Count	Percentage	SQ *p*-value
Gender			0.116
Male	393	49.6%	
Female	400	50.4%	
Age			0.917
12–15 years	204	25.7%	
16–18 years	589	74.3%	
Grade			0.866
Middle School	210	26.5%	
High School	583	73.5%	
Residence			0.808
Rural	264	33.3%	
Urban	529	66.7%	
Boarding			0.351
Yes	287	36.2%	
No	506	63.8%	

### 3.2 Measurement tools

#### 3.2.1 Social ecological risk assessment questionnaire (SERAQ)

Cumulative ecological risk was measured using the SERAQ for adolescents, adapted by Wang J. J. et al. (2024). This scale includes 25 items, such as “My parents often scold me over very minor matters,” with reverse scoring applied to items 6, 7, 11–14, and 20–25. The scale uses a 5-point Likert scale, ranging from 1 (completely agree) to 5 (completely disagree). In line with the cumulative risk perspective, the present study used the overall SERAQ score to represent adolescents’ cumulative exposure to ecological risks rather than comparing the relative associations of individual risk domains. Higher scores indicate a higher level of exposure to CER. The Chinese version of the SERAQ has been validated for reliability and validity in the Chinese student population (Wang J. J. et al., 2024). In this study, the Cronbach’s alpha for the SERAQ was 0.959, indicating good reliability, and the KMO value was 0.989, with a significant Bartlett’s test of sphericity (*p* < 0.001).

#### 3.2.2 Dysfunctional beliefs and attitudes about sleep scale (DBAS-16)

Dysfunctional beliefs and attitudes about sleep were measured using the DBAS-16 developed by Morin et al. (2007). This scale contains 16 items, such as “I need to sleep for 8 h to feel energized and in good condition during the day.” The scale uses a 5-point Likert scale, ranging from 1 (strongly disagree) to 5 (strongly agree). Higher scores indicate a stronger endorsement of DBAS. The Chinese version of the DBAS-16 has been validated for reliability and validity in the Chinese student population (Wang et al., 2025). In this study, the Cronbach’s alpha for the DBAS-16 was 0.942, demonstrating good reliability, and the KMO value was 0.981, with a significant Bartlett’s test of sphericity (*p* < 0.001).

#### 3.2.3 Kessler psychological distress scale (K10)

Psychological distress was measured using the K10, developed by Andrews and Slade (2001). This scale includes 10 items, such as “I feel tired without any reason.” The scale uses a 5-point Likert scale, ranging from 1 (never) to 5 (always). Higher scores indicate greater PD. The Chinese version of the K10 has been validated for reliability and validity in the Chinese student population (Deng et al., 2024). In this study, the Cronbach’s alpha for the K10 was 0.899, demonstrating good reliability, and the KMO value was 0.952, with a significant Bartlett’s test of sphericity (*p* < 0.001).

#### 3.2.4 B-Pittsburgh sleep quality index scale (B-PSQI)

Sleep quality was measured using the Brief Pittsburgh Sleep Quality Index (B-PSQI) developed by Sancho-Domingo et al. (2021). The scale includes six items assessing bedtime, wake time, actual sleep duration, and sleep-related experiences during the past month. Sleep efficiency was calculated as actual sleep time divided by total time in bed and then multiplied by 100. Actual sleep time was obtained from Item 4, and total time in bed was calculated as wake time in Item 2 minus bedtime in Item 1. The total B-PSQI score was calculated by summing Item 3, Item 4, Item 5, Item 6, and the sleep efficiency score. Higher total scores indicate better sleep quality. The Chinese version of the B-PSQI has been validated for reliability and validity in Chinese student populations (Gong et al., 2024). In this study, Cronbach’s alpha for the B-PSQI was 0.781, the KMO value was 0.788, and Bartlett’s test of sphericity was significant (*p* < 0.001).

The translation-back translation procedure described by Brislin (1969) was applied to adapt the questionnaire both linguistically and culturally. Two independent translators worked separately on the initial translation from English to Chinese. One had expertise in educational psychology and was proficient in both Chinese and English, while the other was a linguist with in-depth knowledge of Chinese language usage. Their translations were then synthesized into a single version after detailed comparison and discussion. This version was later translated back into English by a bilingual expert uninvolved in earlier stages. Any inconsistencies between the back-translated and original English versions were examined to detect conceptual or semantic deviations. Necessary refinements were introduced to preserve content equivalence. A preliminary test with 20 adolescents was then carried out to assess the clarity and cultural appropriateness of the items. Participant feedback was used to refine vague or unnatural expressions. The finalized version incorporated all necessary adjustments, ensuring the instrument’s suitability for the Chinese adolescent population.

### 3.3 Statistical analysis

We utilized SPSS 26.0 and AMOS 24.0 to carry out all statistical procedures. Reliability and construct validity for the measurement instruments were examined using Cronbach’s alpha, composite reliability, and average variance extracted (AVE). Descriptive analyses provided an overview of the data, while skewness and kurtosis statistics were used to inspect distributional characteristics. Pearson correlations were computed to assess bivariate associations between core variables. Model fit was evaluated through confirmatory factor analysis (CFA). To evaluate the potential influence of common method bias and variable redundancy, we applied Harman’s single-factor test and checked for multicollinearity. For mediation analysis, we employed Model 4 of the PROCESS macro with bootstrapping (5,000 resamples). CER and DBAS were treated as predictor variables, PD as the proposed indirect pathway variable, and SQ as the outcome measure. The demographic variables were statistically adjusted as covariates. A 95% confidence interval (CI) was used to evaluate the statistical evidence for the estimated indirect association (Preacher and Hayes, 2008). When the bias-corrected CI excluded zero, the estimated indirect association was considered statistically different from zero at the 0.05 level (Hayes and Rockwood, 2017).

## 4 Results

### 4.1 Construct reliability and validity

Internal consistency was evaluated using composite reliability and Cronbach’s alpha coefficient (see Table 2). According to the recommendations of Hair et al. (2021), composite reliability values between 0.60 and 0.70 are considered acceptable, and values between 0.70 and 0.90 indicate good reliability. A Cronbach’s alpha coefficient above 0.70 is considered to meet the standard (Leguina, 2015). The results in Table 2 show that the composite reliability and Cronbach’s alpha coefficients for all scales met the standards, indicating high internal consistency. Next, convergent validity was further assessed. Convergent validity was evaluated using AVE, with values greater than 0.50 considered acceptable (Leguina, 2015). The results show that the AVE for all constructs exceeded 0.50, indicating good consistency among the measurement indicators.

**Table 2:** Reliability and validity.

Constructs	Cronbach’s alpha	Composite reliability	AVE
SQ	0.781	0.784	0.604
CER	0.959	0.960	0.505
DBAS	0.942	0.944	0.536
PD	0.899	0.905	0.525

### 4.2 Descriptive statistics and correlation analysis

Table 3 presents the descriptive statistics and Pearson correlation results for the main study variables. To provide a comprehensive overview of the central tendency and distribution characteristics of each latent variable, the mean (M), standard deviation (SD), skewness (SK), and kurtosis (Kur) are reported. The results show that the skewness and kurtosis values for all variables fall within acceptable ranges, indicating that the data generally meet the assumption of normal distribution.

**Table 3:** Descriptive statistics and correlation analysis.

Constructs	*N*	M ± SD	SK	Kur	CER	DBAS	PD	SQ
CER	793	72.752 ± 12.209	0.158	0.243	1			
DBAS	793	48.069 ± 8.340	−0.080	−0.050	0.248^***^	1		
PD	793	29.463 ± 5.125	0.033	−0.143	0.445^***^	0.352^***^	1	
SQ	793	85.396 ± 9.874	−0.136	−0.078	−0.322^***^	−0.356^***^	−0.449^***^	1

^***^p < 0.001.

In terms of correlation results, CER was significantly negatively associated with SQ (*r* = −0.322, *p* < 0.001), and DBAS also showed a significant negative association with SQ (*r* = −0.356, *p* < 0.001), providing preliminary support for H1 and H2. Additionally, CER (*r* = 0.445, *p* < 0.001) and DBAS (*r* = 0.352, *p* < 0.001) were significantly positively correlated with PD, while PD was significantly negatively correlated with SQ (*r* = − 0.449, *p* < 0.001).

### 4.3 Common method bias

To assess the potential issue of common method bias, this study employed Harman’s single-factor test. The results showed that four factors had eigenvalues greater than 1, and the first factor accounted for 29.311% of the total variance. This is below the commonly accepted threshold of 40% (Podsakoff et al., 2003), indicating that common method bias was not a serious concern in this study.

### 4.4 Multicollinearity diagnosis

To ensure the stability of the model and the validity of the interpretations, this study conducted a detailed diagnosis of multicollinearity among the independent variables. Tolerance and VIF values were calculated using SPSS to evaluate potential multicollinearity issues. As shown in Table 4, all tolerance values were above 0.1, and all VIF values were below 3.3. According to the criteria suggested by Hair et al. (2019), these results indicate that there are no severe multicollinearity problems. Therefore, the impact of multicollinearity on the present study is within an acceptable range.

**Table 4:** Multicollinearity diagnostics for structural model.

Constructs	Tolerance	VIF
CER	0.793	1.262
DBAS	0.866	1.155
PD	0.740	1.351

### 4.5 Confirmatory factor analysis

To assess the quality of the measurement model, this study conducted a CFA to test whether the factor structure of the questionnaire aligned with theoretical expectations. This step was essential to ensure that the instruments validly captured the intended latent constructs. As shown in Table 5, the CFA results indicated that the proposed model demonstrated a good overall fit to the data, with all key indices meeting or exceeding commonly accepted thresholds for model adequacy.

**Table 5:** Model fit indices.

Fit index	Recommended threshold	Final model result
CMIN/DF	<5	1.015
RMSEA	<0.05	0.004
GFI	>0.9	0.940
AGFI	>0.9	0.935
CFI	>0.9	0.999
IFI	>0.9	0.999
TLI	>0.9	0.999

### 4.6 Statistical mediation analysis

After controlling for demographic variables (gender, age, grade level, residence location, and boarding status), the regression results for the mediation model are presented in Table 6. CER was significantly positively associated with PD (*β* = 0.445, *p* < 0.001), and PD was significantly negatively associated with SQ (*β* = −0.380, *p* < 0.001). The direct association between CER and SQ also remained significant (*β* = −0.155, *p* < 0.001). Similarly, DBAS was significantly positively associated with PD (*β* = 0.351, *p* < 0.001), and PD was significantly negatively associated with SQ (*β* = −0.372, *p* < 0.001). The direct association between DBAS and SQ also remained significant (*β* = −0.226, *p* < 0.001). These results suggest that both CER and DBAS are indirectly related to lower SQ among adolescents by being associated with higher levels of PD. At the same time, both variables show significant direct associations with SQ.

**Table 6:** Mediation model of PD between CER, DBAS, and SQ (*N* = 793), controlling for gender, age, grade, residence, and boarding status.

Outcome variable	Predictor variable	*β*	SE	*T*	Bootstrap 95% CI		*R^2^*	*F*
					LLCI	ULCI		
PD	CER	0.445^***^	0.032	13.992^***^	0.383	0.508	0.202	33.250
	Gender	0.096	0.068	1.418	−0.037	0.229		
	Age	0.032	0.074	0.432	−0.113	0.177		
	Grade	−0.045	0.073	−0.618	−0.189	0.099		
	Residence	0.099	0.068	1.458	−0.034	0.233		
	Boarding Status	0.014	0.068	0.211	−0.118	0.147		
SQ	CER	−0.155^***^	0.035	−4.394^***^	−0.224	−0.085	0.224	32.375
	PD	−0.380^***^	0.035	−10.797^***^	−0.449	−0.311		
	Gender	−0.076	0.067	−1.129	−0.207	0.056		
	Age	0.046	0.073	0.636	−0.097	0.190		
	Grade	−0.027	0.072	−0.372	−0.169	0.115		
	Residence	−0.005	0.067	−0.070	−0.137	0.127		
	Boarding Status	0.096	0.067	1.439	−0.035	0.227		
PD	DBAS	0.351^***^	0.034	10.440^***^	0.285	0.417	0.125	18.718
	Gender	0.024	0.067	0.355	−0.108	0.155		
	Age	0.059	0.077	0.763	−0.093	0.211		
	Grade	−0.026	0.077	−0.333	−0.177	0.125		
	Residence	0.023	0.072	0.316	−0.118	0.163		
	Boarding Status	0.043	0.071	0.614	−0.095	0.182		
SQ	DBAS	−0.226^***^	0.033	−6.792^***^	−0.291	−0.161	0.250	37.474
	PD	−0.372^***^	0.033	−11.255^***^	−0.437	−0.307		
	Gender	0.076	0.062	1.229	−0.045	0.198		
	Age	0.037	0.072	0.516	−0.104	0.178		
	Grade	−0.056	0.071	−0.787	−0.196	0.084		
	Residence	0.039	0.066	0.592	−0.091	0.169		
	Boarding Status	0.081	0.065	1.245	−0.047	0.210		

^***^p < 0.001.

The mediation effects (as shown in Table 7 and Figure 2) revealed that the total effect of CER on SQ was −0.323, while the direct effect was −0.155. When PD was included as a mediator, the indirect effect was −0.169, accounting for 52.327% of the total effect. Similarly, the total effect of DBAS on SQ was −0.356, and the direct effect was −0.226. The indirect effect via PD was −0.131, accounting for 36.797% of the total effect. These findings provide empirical support for H3 and H4, supporting the statistical indirect associations involving PD in both pathways.

**Table 7:** Indirect associations involving PD.

Effect	Path	*β*	Bootstrap 95% CI		Percentage (%)
			LLCI	ULCI	
Total effect	CER → SQ	−0.323	−0.389	−0.257	100.000
	DBAS → SQ	−0.356	−0.422	−0.290	100.000
Direct effect	CER → SQ	−0.155	−0.224	−0.085	47.987
	DBAS → SQ	−0.226	−0.291	−0.161	63.483
Indirect effect	CER → PD → SQ	−0.169	−0.208	−0.132	52.327
	DBAS → PD → SQ	−0.131	−0.165	−0.098	36.797

**Figure 2:**
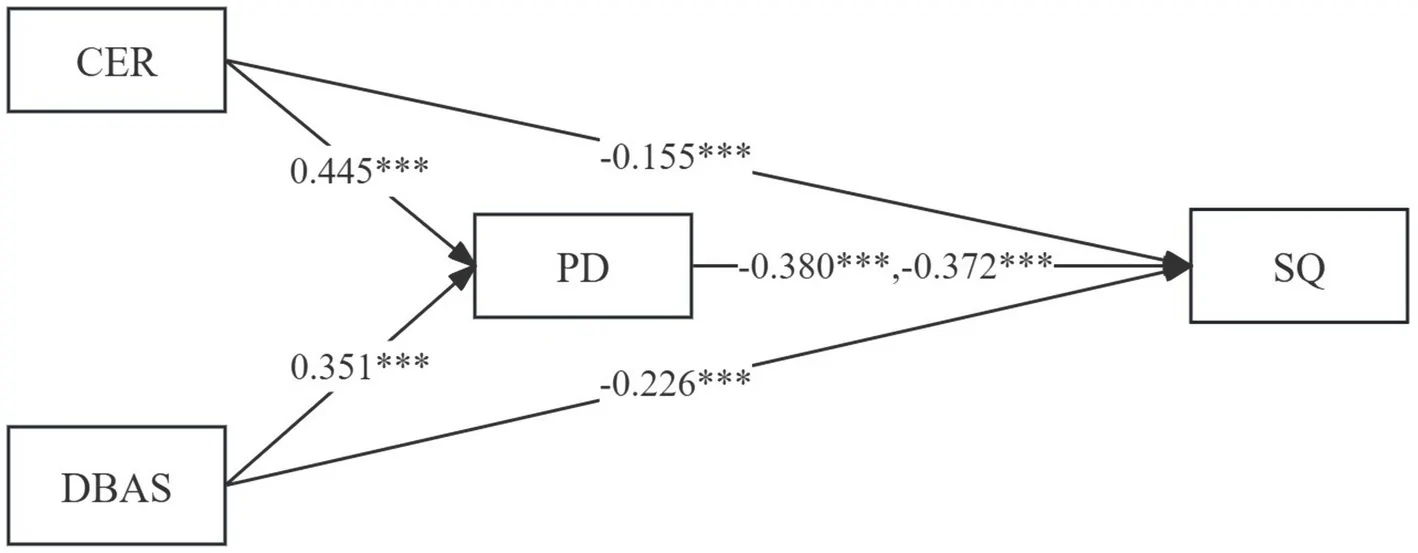
Mediation model diagram. ^***^p < 0.001.

## 5 Discussion

The present research examined the associations of CER and DBAS with SQ and whether PD was statistically involved in these indirect associations. Findings revealed significant negative correlations between CER, DBAS, and PD with SQ. Analyses of indirect associations indicated that PD was statistically involved in the associations of both CER and DBAS with SQ. These patterns highlight the relevance of PD for understanding adolescent sleep-related challenges and may provide empirical references for future research and practice.

The study found a significant negative association between CER and adolescents’ SQ, supporting H1. This pattern is consistent with Xie et al. (2024), who reported that higher ecological risk across family, peer, and academic contexts was associated with lower SQ. According to EST, adolescents’ microsystems and mesosystems often contain multiple sources of potential stress (Bronfenbrenner, 1979). These risks do not exist in isolation but tend to accumulate and are linked to higher levels of psychological burden. In such contexts, higher ecological risk is associated with greater psychological tension, heightened vigilance, recurrent negative thinking, and emotional fluctuations, which have also been associated with poor SQ (Zheng et al., 2023). This finding contributes to a more comprehensive understanding of factors associated with adolescent sleep disturbances from the perspective of ecological systems and provides empirical evidence for identifying the broader social and environmental contexts related to poor SQ.

The DBAS were significantly negatively associated with adolescents’ SQ, providing support for H2. This finding is consistent with the results of Peng et al. (2023). According to the SVM (Zubin and Spring, 1977), DBAS can be understood as a cognitive vulnerability trait. Within this framework, DBAS are often associated with elevated emotional arousal, negative psychological states, and sleep difficulties such as trouble falling asleep or frequent awakenings (Peng et al., 2023; Wang et al., 2025). Because cognitive appraisal and emotion regulation are still developing during adolescence, persistent worry, pessimistic anticipation, and heightened self-monitoring may be particularly relevant to the association between DBAS and SQ (Wang et al., 2025; Zheng et al., 2025). In school environments emphasizing academic performance, rigid beliefs such as “I must sleep well” or “If I do not sleep well, something will go wrong” may coexist with poorer psychological health and SQ (Karsan et al., 2024). In summary, the findings highlight the stable association between DBAS and adolescents’ SQ. The findings suggest that both external stressors and internal cognitive response patterns are relevant factors in relation to adolescent sleep health. Cognitive-based approaches may be relevant considerations in future sleep health–related practice and research.

The present investigation identified a statistically significant indirect association involving PD between CER and SQ, providing support for H3. This result is consistent with previous research (Li et al., 2023; Liu et al., 2024; Wang J. J. et al., 2024; Xu et al., 2024). Adolescents with higher CER often report simultaneous exposure to limited family support, peer rejection, academic demands, and strained community relationships (Bronfenbrenner, 1977). Higher levels of social stressors have frequently been associated with adverse emotional states, including heightened anxiety, low mood, emotional agitation, and social withdrawal. Because emotional regulation capacities are still developing during adolescence, prolonged exposure to multiple stressors may coexist with sustained emotional strain (Sun et al., 2021). Moreover, PD is often closely associated with pre-sleep hyperarousal, somatic discomfort, and perceived difficulties in initiating or maintaining sleep (AlRasheed et al., 2022). Higher levels of PD were associated with greater sensitivity to perceived sleep fluctuations, more negative interpretations of sleep disturbances, and more negative subjective sleep experiences (Duong, 2021). In summary, the findings support the statistical indirect association involving PD between CER and SQ. A comprehensive view of adolescent sleep difficulties emerges when EST is considered alongside emotional regulation approaches, as reflected in the current findings.

The PD was found to be statistically involved in the indirect association between DBAS and SQ, supporting H4. This result is consistent with prior research (Faaland et al., 2023; Peng et al., 2023; Wang et al., 2025). Individuals who endorse such beliefs tend to focus excessively on their sleep performance and set unrealistic expectations. Perceived discrepancies between sleep experiences and expectations were associated with more intense negative emotions, including anxiety, frustration, and disappointment, as well as higher levels of PD. Elevated PD is often associated with greater concerns about sleep failure and a recurring pattern of negative emotional and cognitive responses (Peng et al., 2023). Within this context, DBAS are often accompanied by pre-sleep hyperarousal, anxiety about sleep failure, and persistent worries about sleep (Wang et al., 2025). Higher levels of PD are also associated with greater subjective sleep frustration, perceived insufficient recovery, and lower overall SQ. These emotional experiences and cognitive beliefs may become closely intertwined, forming a relatively stable pattern of association (Duong, 2021; Li et al., 2023). To conclude, the results obtained in this study support the statistical indirect association involving PD between DBAS and SQ. Understanding these psychological associations may inform future research on theoretically grounded interventions.

### 5.1 Implications

#### 5.1.1 Theoretical implications

Grounded in EST and the SVM, this study proposed and tested a statistical indirect association model involving environmental stress, PD, and subjective health experiences. It extends the applicability of these frameworks within adolescent sleep research. Specifically, this study incorporated PD as a central variable involved in the indirect associations among CER, DBAS, and SQ. This finding contributes to the ecological perspective by highlighting the co-occurrence of multiple risk exposures and adds to the theoretical discussion of cognitive vulnerability and emotional dysregulation as described in the SVM. Although previous studies have examined factors associated with sleep disorders from various perspectives, this study integrates external environmental stress and internal cognitive bias within a unified analytical framework. It further examines whether these two factors are jointly associated with adolescents’ SQ and whether PD is involved in these associations. By incorporating multiple psychological dimensions, this model supports a more comprehensive examination of adolescents’ everyday mental health experiences. Taking adolescent psychological development into account, this research identifies significant indirect associations involving PD in relation to patterns of sleep difficulties. These findings provide empirical references for future developmental research in the field of sleep.

#### 5.1.2 Practical implications

From a practical standpoint, the findings may have implications for future efforts related to adolescent sleep health, family support, and psychological services. First, in school-based education, it is recommended to strengthen the identification and support of students with high exposure to risk. For adolescents reporting chronic academic, interpersonal, or family-related stress, structured programs concerning cognitive restructuring, stress management, and emotional regulation may be considered as potential forms of support, although their effectiveness requires evaluation through longitudinal or intervention research. Second, in the context of family education, parents should be encouraged to become more sensitive to adolescents’ emotional responses and sleep conditions. A stable and supportive family communication environment and less neglectful or critical parenting styles may be relevant to adolescents’ cognitive and emotional adjustment. Schools and communities may also collaborate to promote parenting education programs. Home-school collaboration and educational workshops may provide opportunities for parents to learn more about healthy sleep patterns and emotional support for adolescents. Third, at the level of student support, adolescents should be guided to strengthen self-awareness and self-regulation. Future student-support programs may consider adolescents’ sleep-related beliefs, sleep routines, lifestyles, self-awareness, and psychological resources when addressing stress-related challenges. Finally, the findings offer policy implications for educational authorities and public health institutions. Effective adolescent mental health planning requires acknowledging the complex associations among environmental stressors, maladaptive thinking patterns, and emotional disturbances. A coordinated approach is needed to promote synergy across school health education, psychological services, and family support policies.

### 5.2 Limitations and future research directions

Although this study yielded preliminary findings regarding the associations among CER, DBAS, PD, and SQ among adolescents. First, this study employed self-report questionnaires to collect data. Although self-report measures are appropriate for assessing subjective beliefs, psychological distress, and perceived sleep quality, collecting all variables from the same respondents at a single time point may introduce common method bias, including social desirability, recall bias, and consistency motives. Such shared method variance may inflate or attenuate the observed correlations and statistical indirect associations. To strengthen the robustness of future analyses, researchers might consider integrating diverse forms of data collected across different sources. In addition to standardized self-report measures, researchers may incorporate parent or teacher evaluations, sleep behavior logs, and records from wearable devices to capture adolescents’ sleep and psychological conditions from multiple perspectives.

Second, the cross-sectional design of this study only allows the examination of associations among CER, DBAS, PD, and SQ, and therefore the temporal ordering among these variables cannot be determined. Although the mediation model suggests that psychological distress may represent an indirect pathway linking ecological risk and dysfunctional sleep-related cognitions with sleep quality, the current design does not allow conclusions regarding causal pathways. Future studies are encouraged to adopt longitudinal designs and conduct follow-up assessments with a subsample of participants across multiple time points to examine changes in CER, DBAS, PD, and SQ and clarify their temporal relationships.

Third, although the B-PSQI collected information on some sleep-related behaviors, including sleep duration, bedtime, and wake time, these indicators were not analyzed separately in the present study. In addition, sleep regularity, social jetlag, weekend catch-up sleep, restorative sleep, and subjective sleep refreshment were not assessed. Future studies should conduct more detailed analyses of specific sleep behaviors and incorporate these additional dimensions to offer a broader insight of the associations among ecological risk, dysfunctional sleep-related beliefs, psychological distress, and adolescent sleep health.

Fourth, this study relied on an overall cumulative ecological risk score and did not separately examine the specific contributions of family-, school-, peer-, and community-related risks. Although the cumulative score captures adolescents’ overall exposure to multiple ecological risks, it may obscure differences across individual risk domains. Future studies should conduct subscale-level analyses to identify which ecological risk factors are most closely linked to psychological distress and sleep quality, thereby providing more targeted implications for intervention.

Fifth, although the study included a relatively large sample, the participants were recruited from selected middle and high schools in China, and most were aged 16–18 years and enrolled in high school. Thus, the applicability of the present results to adolescent populations in other geographical areas, school contexts, and developmental stages might be constrained. Subsequent investigations ought to employ samples that are more varied in geography and demography, so as to test the stability of these outcomes.

Sixth, intervention-based research could be conducted. For instance, the implementation of cognitive restructuring training, emotional regulation skills development, or multidimensional psychological support programs could help to evaluate whether changes in DBAS and PD are associated with changes in SQ. Such efforts may provide more robust empirical evidence for clarifying potential causal relationships.

## 6 Conclusion

Based on EST and the SVM, this study explored the indirect associations among CER, DBAS, PD, and adolescents’ SQ. The findings indicated that CER, DBAS, and PD were all significantly negatively associated with SQ. Moreover, PD was statistically involved in the indirect associations between both risk factors and SQ. Compared with previous studies that typically examined environmental risk or cognitive beliefs separately, this study integrated external ecological stress and internal cognitive bias within a multivariate model that included statistical indirect associations involving PD. This approach identified significant indirect associations involving PD among ecological risk, sleep-related beliefs, and subjective health experiences. These results extend the application of ecological theory in adolescent sleep research and contribute to understanding the psychological factors associated with sleep disturbances. In light of increasing concerns about adolescent sleep problems, this study underscores the value of jointly considering ecological conditions, sleep-related cognitive beliefs, and emotional states when developing future adolescent support programs. It points to the potential value of coordinated school-based education, family support, and individual services in relation to adolescents’ psychological adjustment and sleep health, although their effectiveness requires further evaluation. In summary, this study provides theoretical insight into the multiple psychological factors associated with poor SQ among adolescents and may offer practical references for future multidimensional support and intervention research.

## Data Availability

The raw data supporting the conclusions of this article will be made available by the authors, without undue reservation.

## Footnotes

1
https://www.wjx.cn/
